# Infection with human herpesvirus type 8 and human T-cell leukaemia virus type 1 among individuals participating in a case–control study in Havana City, Cuba

**DOI:** 10.1038/sj.bjc.6600613

**Published:** 2002-11-12

**Authors:** L Fernandez, D Serraino, G Rezza, J Lence, R M Ortiz, T Cruz, S Vaccarella, L Sarmati, M Andreoni, S Franceschi

**Affiliations:** Instituto Nacional de Oncología y Radiobiología, Havana, Cuba; Dipartimento di Epidemiologia, Istituto Nazionale per le Malattie Infettive L. Spallanzani, IRCCS, Rome, Italy; Centro Operativo AIDS, Istituto Superiore di Sanità, Rome, Italy; Unit of Field and Intervention Studies, International Agency for Research on Cancer, Lyon, France; Istituto di Malattie Infettive, Università di Tor Vergata, Rome, Italy

**Keywords:** Cuba, HHV-8, HTLV-1, Kaposi's sarcoma, prevalence, risk factors

## Abstract

Infection with human herpesvirus type 8 and with human T-cell leukaemia virus type-1 shows strong geographic variations. We conducted this study to assess prevalence and risk factors for human herpesvirus type 8 infection in Havana City, Cuba. Information and residual serum samples already collected for a hospital based case–control study were used. A total of 379 individuals (267 males and 112 females; median age=63 years) were evaluated. Antibodies to the lytic antigen of human herpesvirus type 8 were detected by using an immunofluorescence assay, while human T-cell leukaemia virus type-1 serology was performed by means of an ELISA test (alpha Biotech). Overall, 64 subjects (16.9%, 95% confidence interval: 13.1–20.0) were positive for human herpesvirus type 8 antibodies. Human herpesvirus type 8 seroprevalence significantly increased with age (odds ratio=1.9 for ⩾65 *vs* <55 years), and was twice as frequent in blacks than in whites. No association emerged with gender, socio-economic indicators, family size, history of sexually transmitted disease, sexual behaviour. Overall, 16 persons had anti-human T-cell leukaemia virus type-1 antibodies (4.2%, 95% confidence interval: 2.2–6.4). No relationship emerged between human T-cell leukaemia virus type-1 and human herpesvirus type 8 serostatus. The study findings indicate that human herpesvirus type 8 infection is relatively common in Havana City, Cuba, suggesting that Cuba may represent an intermediate endemical area. Sexual transmission does not seem to play a major role in the spread human herpesvirus type 8 infection.

*British Journal of Cancer* (2002) **87**, 1253–1256. doi:10.1038/sj.bjc.6600613 www.bjcancer.com

© 2002 Cancer Research UK

## 

Since its discovery in 1994, the human herpesvirus type 8 (HHV-8) – the causal agent of Kaposi's sarcoma (KS) – has been documented in virtually every form of KS, i.e., in the classic, or Mediterranean type; in the endemic, or African type; and in the AIDS-associated type (Chang *et al*, 1994; IARC Working Group on the Evaluation of Carcinogenic Risks to Humans, 1997). Many cross-sectional investigations conducted in different geographic areas have put in evidence that the prevalence of HHV-8 infection mirrors incidence rates of AIDS-unrelated KS. The lack of standardised serological assays against HHV-8 antigens still represents a major drawback for the comparison of findings from different investigations (IARC Working Group on the Evaluation of Carcinogenic Risks to Humans, 1997; Schatz *et al*, 2001).

The prevalence of HHV-8 infection in Caribbean populations has been little investigated, and, in these areas, incidence rates for KS are not available (Lennette *et al*, 1996; Chatlynne and Ablashi, 1999). About 8% of male blood donors, aged 50 years or older, were seropositive for HHV-8 infection in Jamaica (Manns *et al*, 1998), while an investigation conducted among Haitian women migrated to the United States showed that 29% of them were infected with HHV-8 (Goedert *et al*, 1997).

To study the distribution of HHV-8 infection, we took advantage of a case–control study on oral cancer conducted in Havana City, Cuba, part of a wider study coordinated by the International Agency for Research on Cancer, Lyon (Garrote *et al*, 2001). In addition we examined the prevalence of seropositivity for HHV-8 antibodies and certain potential correlates of infection, such as socio-demographic characteristics, history of sexually transmitted diseases (STD) and sexual behaviour, and infection with human T-cell leukaemia virus type-1 (HTLV-1).

## METHODS

This seroepidemiological investigation took advantage of residual serum samples and from information already collected in a hospital-based case–control study (Garrote *et al*, 2001). The first 200 patients newly diagnosed with cancer of the oral cavity or of the oropharynx diagnosed between April 1996 and July 1999 in the Instituto Nacional de Oncología y Radiobiología, Havana City, Cuba, represented the cases of the original study. These 200 cases were histologically confirmed and they did not receive any prior local or systemic cancer treatment. During the same period, an equal number of controls were identified from the same hospitals of the cases and they were matched to cases by sex and age (in quinquennia). The controls had no history of, or current suspicion of, cancer of the oral cavity or oropharynx. With respect to eligible reasons of hospital admission for control subjects, diseases associated positively or negatively with the known or suspected risk factors for cancer of the oral cavity or oropharynx (e.g., heavy smoking or alcohol abuse) were excluded. Cases and controls consented to participate voluntarily in the study and they were in physical and mental conditions to give reliable answers to the questionnaire (Garrote *et al*, 2001).

Potential infection with human immunodeficiency virus (HIV) was investigated, though none of the enrolled individuals were aware of having acquired HIV infection. However, neither the cases nor the controls were tested for HIV antibodies.

Residual sera for assessing the presence of antibodies against HHV-8 were not available for nine cases and 12 controls (median age: 62 years) out of the 400 individuals originally enrolled in the case–control study. Thus, 379 individuals (267 males and 112 females; median age=63 years) constituted the study group for the present investigation.

Antibodies to the lytic antigen of HHV-8 were detected by using an immunofluorescence assay (IFA) based on BCBL-1 (body cavity B-cell lymphomas) cell line (obtained through the AIDS Research and Reference Reagent Program, Division of AIDS, National Institutes of Health, from Drs M McGrath and D Ganem) and on BCP-1 cell line. For the purpose of this study, titres of 1 : 20 or more were considered positive. Details on the assay were previously published (Andreoni *et al*, 1999; Rezza *et al*, 1999; Schatz *et al*, 2001). HTLV-1 serology was performed by means of an ELISA test (alpha Biotech). ELISA-positive findings were all confirmed by means of Western blot technique (GeneLab).

### Statistical analysis

At univariate analysis, the chi-square test for trend was used to test the statistical significance between ordered categorical variables and HHV-8 seropositivity (Armitage and Berry, 1987). Odds ratios (OR) and their 95% confidence intervals (CI) were used to assess the association between HHV-8 seropositivity and various characteristics and exposures by means of unconditional multiple logistic regression (Breslow and Day, 1980).

## RESULTS

Overall, 64 individuals (47 men and 17 women, median age=63 years) (16.9%, 95% CI: 13.1–20.0) were positive for HHV-8 antibodies. Prevalence of HHV-8 infection was similar among the 191 patients with cancer (17.3%) and the 188 controls (16.5%) (*P*=0.95) (data not shown in tables).

As listed in Table 1

**Table 1:**
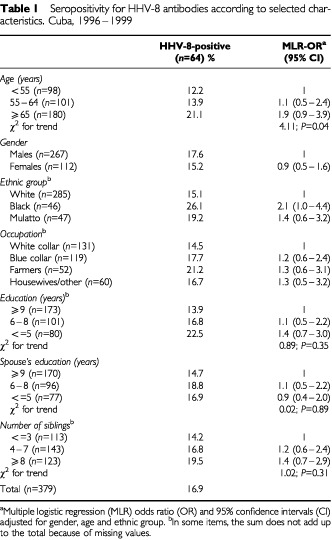
Seropositivity for HHV-8 antibodies according to selected characteristics. Cuba, 1996–1999

, HHV-8 seroprevalence significantly increased with the increase of age (χ^2^ for trend, *P*=0.04), ranging from 12.2% in subjects younger than 55 years to 21.1% in those aged 65 years or older (OR=1.9). HHV-8 infection was twice as frequent in blacks than in whites (95% CI: 1.0–4.4), whereas males and females presented similar seropositivity rates (17.6 and 15.2%, respectively) (Table 1).

All associations described below were evaluated after adjustment for age, gender, and ethnic group. None of the socio-economic indicators (e.g., education: ⩽5 years *vs* ⩾9, OR=1.4, 95% CI: 0.7–3.0), and family size indicators (i.e., ⩾8 siblings *vs* ⩽3, OR=1.4, 95% CI=0.7–2.9) turned out to be associated with HHV-8 seropositivity (Table 1).

Seropositivity for HHV-8 antibodies was not associated with history of STD, neither with sexual behaviour, such as age at first intercourse or lifetime number of sexual partners (Table 2

**Table 2:**
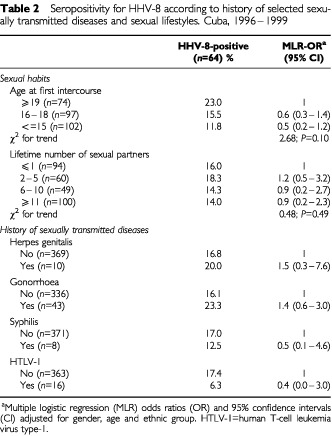
Seropositivity for HHV-8 according to history of selected sexually transmitted diseases and sexual lifestyles. Cuba, 1996–1999

). These results did not change when the analysis was separately conducted among males or females (data not shown in tables).

Overall, 16 of these 379 examined persons had anti-HTLV-1 antibodies (4.2%, 95% CI: 2.2–6.4). No relationship emerged between HTLV-1 and HHV-8 serostatus, though a non-statistically significant inverse association was recorded (Table 2).

## DISCUSSION

The prevalence of HHV-8 infection in this group of population living in Havana City, Cuba, appeared to be intermediate between those reported in areas where KS is rare, like northern Europe and North America, and areas where KS is more common, like southern Italy (Geddes *et al*, 1995; Parkin *et al*, 1997). Interestingly, the findings of this seroprevalence investigation suggest that sexual habits, particularly sexual promiscuity, are not among the main determinants of HHV-8 infection in Cuba. The lack of association between HHV-8 and HTLV-1 infections also points to a minor role of the sexual route of transmission for these two viral infections in the general population of Cuba.

Age and ethnic group turned out to be the strongest determinants of HHV-8 infection. An increase in prevalence of HHV-8 infection with ageing has been consistently reported (IARC Working Group on the Evaluation of Carcinogenic Risks to Humans, 1997; Plancoulaine *et al*, 2000). In comparison with population groups of the same age (i.e., with a median age of 63 years), the HHV-8 prevalence recorded in this Cuban population appears to be higher than that observed in Asian countries (IARC Working Group on the Evaluation of Carcinogenic Risks to Humans, 1997; Chatlynne and Ablashi, 1999), and similar to that observed among cancer patients aged 50 years or older in the South of Italy (i.e., 16.0%) (Serraino *et al*, 2000; Vitale *et al*, 2001).

Black ethnic origin was associated with a two-fold higher risk of being infected with HHV-8 than white origin, whereas mulatto ethnic origin was at intermediate risk (i.e., 1.4-fold). Such observation mirrors findings from studies of other γ-herpesviruses conducted in North America, showing higher prevalence of antibodies against Epstein-Barr virus (EBV) in black, as compared to white Americans (Hallee *et al*, 1974), though explanations for these findings are still unknown.

No association emerged, in this study, between HHV-8 and demographic indicators, such as occupation and education, suggesting that the spread of HHV-8 in the general population of Havana City, Cuba, is not influenced by socio-economic differences. In this connection the lack of association between HHV-8 seropositivity and family size may be noted. None of the sexual behaviours investigated, and none of the self-reported STD were associated with HHV-8 infection. In accordance to other studies, these findings tend to exclude a major role of sexual transmission of HHV-8 in HIV-negative heterosexual adults (IARC Working Group on the Evaluation of Carcinogenic Risks to Humans, 1997).

Sixteen out of the 379 study subjects (4.2%) were positive for HTLV-1 antibodies. Such prevalence was slightly lower than those observed in other Caribbean islands where HTLV-1 antibodies are usually recognised in up to 15% of adults (Blattner *et al*, 1982; Yoshida, 1999). Low prevalence for, or even lack of infection with HTLV-I was found in other studies conducted on blood donors in Cuba, some of which did not apparently discriminate between HTLV-1 and HTLV-II (Hernandez Ramirez *et al*, 1991; Silva Cabrera *et al*, 1997). However, the report of sporadic cases of HTLV-1-associated with tropical spastic paraparesis suggests that both the spread of HTLV-1 and major route of transmission in Cuba need to be better investigated (Estrada *et al*, 1995). The lack of association found in this investigation between HHV-8 and HTLV-1 infections suggests that these viruses do not share similar transmission modalities. This fact is little surprising since, in endemic areas, HTLV-1 is mainly transmitted from mother to child by breast feeding, or through infection via contaminated blood (IARC Working Group on the Evaluation of Carcinogenic Risks to Humans, 1996).

In conclusion, the overall findings show that HHV-8 infection is relatively common among older adults living in Havana City, Cuba, suggesting that Cuba represents an intermediate endemical area. HHV-8 and HTLV-1 infections are likely to spread with different routes, and sexual transmission does not seem to play a major role in the spread of HHV-8 infection in Cuba.
